# The effect of nutritional interventions involving dietary counselling on gastrointestinal toxicities in adults receiving pelvic radiotherapy – A systematic review

**DOI:** 10.1002/jmrs.531

**Published:** 2021-07-20

**Authors:** Lauren Andreou, Tracy Burrows, Yolanda Surjan

**Affiliations:** ^1^ School of Health Sciences College of Health, Medicine and Wellbeing The University of Newcastle Callaghan New South Wales Australia; ^2^ Hunter Medical Research Institute New Lambton Heights New South Wales Australia

**Keywords:** diarrhoea, dietary intervention, diet, gastrointestinal toxicity, pelvic radiotherapy

## Abstract

Gastrointestinal (GI) toxicities are common in patients receiving radiotherapy (RT) to the pelvis. This systematic review aims to evaluate the effectiveness of nutritional interventions involving dietary counselling (DC) on GI toxicities in patients receiving pelvic RT. The search method entailed two phases to retrieve studies. Articles from a previous Cochrane review by Lawrie et al. 2018 were assessed for inclusion. An updated systematic search was then conducted to retrieve articles published between 2013 and 2020 from five electronic databases (MEDLINE, EMBASE, CINAHL, CENTRAL and Scopus). The inclusion criteria entailed randomised controlled trials involving adults ≥18 years, undergoing curative pelvic RT, receiving a nutritional intervention involving DC with or without supplements. DC was defined as written or face‐to‐face dietary advice provided before or during RT. Outcomes included GI toxicities reported by validated assessment tools. The Academy of Nutrition and Dietetics Quality Criteria Checklist was utilised to assess quality and risk of bias. Of 1922 studies retrieved, 12 articles encompassing 11 individual RCTs were included. Seven studies included a supplement in addition to DC. Supplements included probiotics, prebiotics, probiotic + soluble fibre, high protein liquid supplement and fat emulsion. Of the 11 studies, one involved individualised DC, and the remaining studies prescribed consumption or avoidance of fats, fibre, lactose, protein and FODMAP. The most common toxicities reported were diarrhoea (*n* = 11), pain/cramping (*n* = 9) and bloating/flatulence (*n* = 5). Three studies stated an improvement in diarrhoea incidence. Results varied between studies. Further quality studies are required to assess the effectiveness of DC, in particular individualised DC on GI toxicities in patients receiving pelvic RT.

## Graphical abstract

Gastrointestinal toxicities are common in patients receiving radiotherapy to the pelvis. This systematic review aims to evaluate the effectiveness of nutritional interventions involving dietary counselling on gastrointestinal toxicities in patients receiving pelvic RT.

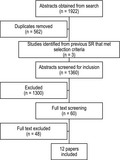

## Background

Gastrointestinal (GI) toxicities are common in patients receiving radiotherapy (RT) to the pelvic area. Commonly occurring GI toxicities include diarrhoea, constipation, vomiting, nausea and pain.^1^ Toxicities often reduce patients’ appetite and the digestive system’s ability to function efficiently, and in some patients, it leads to malnutrition, pain and discomfort during digestion. Additionally, radiotherapy‐induced morbidity, which results from treatment toxicities, may compromise a patient’s nutritional status and negatively impact their quality of life (QOL), both during and after treatment.^1^, ^2^, ^3^

Toxicity to the GI tract is usually a result of an inflammatory response due to repetitive radiation injury to the small and large intestines, exacerbated by the gut’s rapid cell turnover rate.^4^ The inflammatory response leads to increased susceptibility of the bowels to radiation damage, which can reduce the absorptive surface area available and lower enzyme activity. The rate of food passing through the bowels increases, often resulting in essential nutrient and water malabsorption.^5^ As many patients also receive chemotherapy concurrently with RT, toxicities may be further exacerbated as some chemotherapy agents also cause damage to the bowel’s epithelium.^6^

Toxicities due to radiation do not always present immediately.^7^ The prescription length of curative pelvic RT ranges from four to nine weeks.^8^, ^9^ Acute toxicities, such as diarrhoea, dysuria and nausea, often develop circa after two to three weeks of RT and continue to occur for several weeks or months following treatment completion.^7^, ^10^, ^11^ Toxicities can continue to be present due to radiotherapy’s ongoing elimination of cancer cells weeks beyond the completion of RT. The literature commonly refers to toxicities present during treatment or up to 90 days as short term or acute, although toxicities present after three months post‐treatment as chronic or late.^1^, ^12^ Chronic toxicities can include changes in bowel habits, proctitis and incontinence.^1^, ^11^ The National Cancer Institute (USA) Common Terminology Criteria for Adverse Events (CTCAE) is one of the most commonly used tools amongst clinicians for toxicity assessment and grading.^13^ In CTCAE version 3.0 update, the National Cancer Institute moved away from applying a predetermined time‐based classification on toxicities due to each toxicity uniqueness and the nature of current multimodality treatments. Therefore, there is no globally accepted time‐based definition of acute versus chronic toxicities, and the distinction between acute and chronic toxicities often varies between publications.^14^ In addition to CTCAE, a range of tools can be used to assess and grade toxicities. These tools often rely on patients’ self‐reporting and are subjective. For appropriate management of care, both during and after the course of treatment, it is essential that patients are routinely assessed for toxicities by the team of health professionals.^15^

Due to GI toxicities and the disposition of the disease itself, many patients experience weight loss (WL) and malnutrition as their oral intake decreases.^16^ Changes in an individual’s body weight and composition can compromise treatment delivery accuracy. Variations in the external body contour can influence dose distribution as the effective beam path length changes.^17^ This alteration can result in increased dose received by healthy tissues and organs at risk (OARs), thus increasing toxicity risk.^17^, ^18^, ^19^ Changes in the body contour in excess of 1cm or more may prompt treatment replanning, requiring additional time and resources.^17^

The adverse effects of treatment, such as fatigue, pain and toxicities, in addition to the mentally challenging aspect of a cancer diagnosis, can have a significant negative impact on a patient’s QOL during their treatment.^20^ This includes QOL components such as physical, emotional and cognitive functioning. Survival outcomes and tumour response are prominent measures, yet it is also important to consider preserving and maintaining a patient’s QOL.^20^ The EORTC (European Organisation for Research and Treatment of Cancer) Quality of Life of Cancer Patients (QLQ‐C30) tool is the most common instrument used to assess health‐related QOL in clinical oncology trials.^20^ This validated tool is a 30‐item questionnaire that covers global health status, five functional scales (physical, role, cognitive, emotional and social) and nine symptom scales/items (fatigue, nausea/vomiting, pain, dyspnoea, insomnia, appetite loss, constipation, diarrhoea and financial difficulties). Although the tool was initially developed as a QOL assessment tool, the symptoms component, especially within the site‐specific submodules, also allows it to be suitably used to report a number of GI toxicities.

Several studies suggest that the provision of nutritional interventions can decrease toxicities, limit WL and maintain positive QOL.^2^, ^16^, ^21^ The Dietitians Association of Australia (DAA) practice guidelines recommendations include ‘All patients receiving radiation therapy to the GI tract or head and neck area should be screened for nutritional risk and/or referred to the dietitian for nutrition support’.^22^ ^(p.318)^ The guidelines also suggest that patients meet at least fortnightly with a dietitian for monitoring and management during treatment and until approximately six weeks post‐RT.^22^ However, within Australia, protocols, procedures and the implementation of the DAA guidelines likely vary between radiation oncology departments. Nutritional screening, assessments or interventions should be ideally completed by dietetic personnel. However, this is not always achievable as dietetic professionals are not always readily accessible in radiation oncology departments. This may be due to lack of resources, often seen in skill shortage areas or smaller departments in regional or rural areas.^23^ This review aims to assess and evaluate current nutritional interventions (NIs) that involve dietary counselling (DC) and their effect on GI toxicities, WL and QOL arising in patients receiving pelvic RT.

## Method

### Search

A systematic literature search was conducted to source relevant articles that assessed the effect of dietary counselling nutritional interventions on GI toxicities in adults receiving RT to the pelvic region. The search consisted of two phases. Phase 1 involved examining of studies from a previous Cochrane review (Lawrie et al.),^24^ which included a broad range of preventive interventions only (not exclusively nutritional) published from database inception to November 2017, excluding interventions to treat acute GI toxicities. Both included and excluded articles were assessed for possible inclusion in the current review for any studies meeting the current reviews criteria (i.e. nutritional interventions involving a dietary counselling component). Phase 2 involved a systematic database using an updated search strategy search for articles published from 2013 to 2020, from five electronic databases, including MEDLINE, EMBASE, CINAHL, CENTRAL and Scopus. Due to the perpetually evolving development and advancement of RT techniques and technologies, this date range was selected as recent studies were preferred to ensure that the sample RT techniques and modalities are currently utilised in practice. The systematic review search protocol was registered with PROSPERO (CRD42020182760).

### Screening and study selection

The search results were managed using online systematic review management software, Covidence (© 2018 Veritas Health Innovation Ltd). Titles and abstracts were screened independently by two reviewers (LA & KB) for inclusion. The same reviewers conducted the full‐text screening. Any discrepancies were referred to a third reviewer. Interventions based on pharmaceuticals were excluded. The inclusion and exclusion criteria are listed in Table 1.

**Table 1:** Inclusion and exclusion criteria.

	Inclusion	Exclusion
Participants	Adults ≥18 years Any gender Receiving pelvic RT or CRT with curative intent Primary cancer within the pelvis (prostate, bladder, colorectal or gynaecological)	<18 years Receiving palliative treatment Medically diagnosed GI conditions that may impact toxicities (e.g. inflammatory bowel disease, coeliac and stoma) Tube‐feeding, gastrostomy feeding and parenteral nutrition
Intervention	Dietary counselling before or during RT, with or without ONS also prescribed Before or during RT Individual or group Written, face‐to‐face, phone or online	ONS only with nil dietary advice Pharmaceutical interventions
Comparison	Usual care	n/a
Control	Standard or usual care Habitual diet	No comparison group
Outcomes	Primary: GI outcomes (long or short term) Reported using validated tools or as part of QOL questionnaire QOL	n/a
Study type	Randomised control trial	Case study, noncontrolled or nonrandomised studies
Publication year	Phase 1: Cochrane review by Lawrie et al.^24^: database inception – 2017 Phase 2: database update 2013 – 2020	

CRT, chemoradiation therapy; GI, gastrointestinal; ONS, oral nutritional supplements; QOL, quality of life; RT, radiation therapy.

### Data synthesis

A meta‐analysis was not possible due to the variety of interventions and assessment tools used to report GI toxicity outcomes. Therefore, results are presented in a narrative form. For the purpose of this review, the term ‘individualised dietary counselling’ refers to nutritional or diet advice that has been personally tailored to the individual patient, as opposed to generic dietary recommendations that do not consider patient‐specific symptoms or nutritional requirements. Due to variation in GI toxicities reported and terms used, the research team reviewed the individual outcomes and grouped them into 16 umbrella outcomes, with similar toxicities being grouped together. For example, the ‘bloating/flatulence’ group involved all outcomes reported as bloating, flatulence and intestinal gas.

### Quality

Two independent reviewers (LA & KB) assessed study quality and risk of bias. A third reviewer assessed any discrepancies. The Academy of Nutrition and Dietetics Quality Criteria Checklist for Primary Research tool was utilised.^25^ The checklist assessed ten domains of quality and bias. Articles were designated a score of positive, neutral or negative.

## Results

### Study selection

This review identified 12 articles on 11 studies published between 2005 and 2019. Three articles were retrieved from examining studies of the previous Cochrane review by Lawrie et al. that met the selection criteria,^26^, ^27^, ^28^ and nine articles were sourced from the systematic database search.

**Figure 1:**
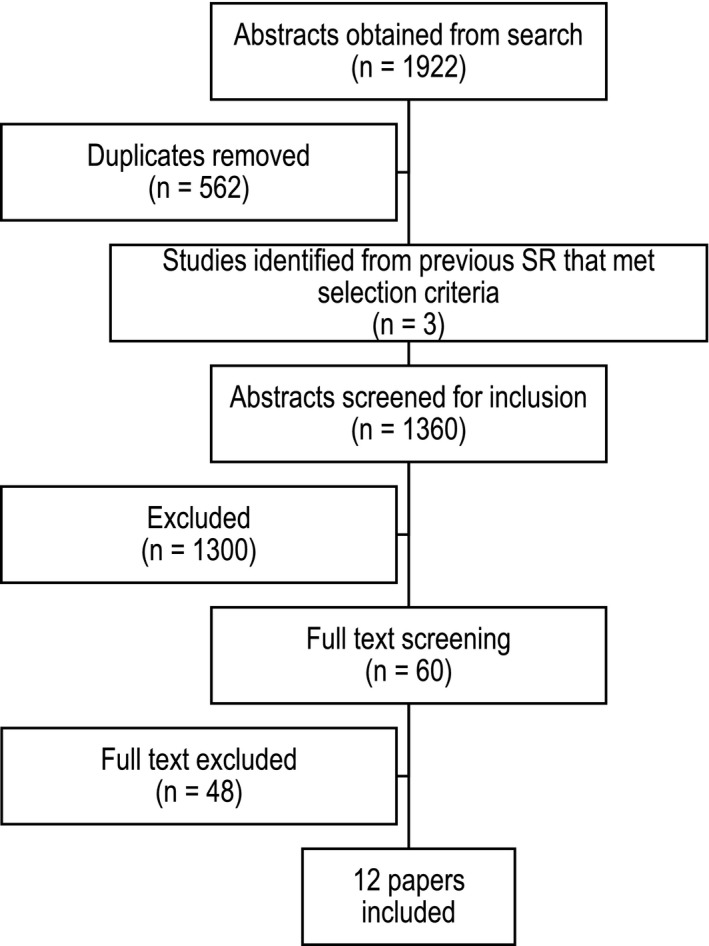
PRISMA study selection flow diagram.

One study had short‐ and long‐term outcomes published across two articles.^26^, ^29^ The systematic review study flow is shown in Figure 1 and reported as per PRISMA guidelines.^30^

### Bias and quality

Study quality assessment results rated six studies of positive quality^5^, ^26^, ^29^, ^31^, ^32^, ^33^ and six as neutral.^27^, ^28^, ^34^, ^35^, ^36^, ^37^

### Study characteristics

The included articles originated from seven countries, with Sweden (*n* = 3)^26^, ^29^, ^31^ and U.K. (*n* = 2)^28^, ^33^ being the most common countries of origin.

Five pelvic cancer tumour sites were assessed within the 11 studies: prostate (*n* = 7),^5^, ^26^, ^28^, ^29^, ^31^, ^35^, ^37^ gynaecological (*n* = 7),^5^, ^28^, ^32^, ^33^, ^34^, ^35^, ^36^ colorectal (*n* = 5),^5^, ^27^, ^28^, ^33^, ^35^ bladder (*n* = 2)^28^, ^35^ and ‘other’ (bone sarcoma) (*n* = 1).^35^ Sample sizes ranged between 17 and 246 participants (mean = 106), totalling 1174 participants. The mean age reported was 63.6 years across six studies where the mean age was disclosed.

Overall, the participants’ genders were approximately equal, with the proportion of female patients being 48%. Four articles^26^, ^29^, ^31^, ^37^ were prostate‐specific; thus, 100% of participants were male. Three articles were exclusively gynaecological.^32^, ^34^, ^36^ The remaining studies included both sexes.

### Radiotherapy treatment details

RT was delivered via external beam radiation therapy (EBRT). RT treatment regimens, techniques and dose prescriptions varied amongst the studies (Table 2 ). Some studies included a combination of treatment techniques^31^, ^33^, ^34^; however, five studies did not state the EBRT technique utilised.^5^, ^26^, ^27^, ^28^, ^29^, ^37^ For the articles that reported on modality, 3‐dimensional conformal RT (3DCRT) (*n* = 3),^33^, ^34^, ^36^ conventional 2‐dimensional RT (*n* = 3),^32^, ^34^, ^35^ volumetric modulated arc therapy (VMAT) (*n* = 1),^31^ and intensity modulated radiation therapy (IMRT) (*n* = 2)^31^, ^33^ was used. Prescriptions ranged from 36 to 78 Gy. Some studies involved a boost delivered by brachytherapy, protons or EBRT.^5^, ^26^, ^29^, ^31^, ^33^, ^34^ Where reported, boost prescriptions ranged from 20 to 30 Gy in 2–10 fractions.^26^, ^29^, ^31^

**Table 2:** RT ± chemotreatment characteristics.

Author	Primary site(s)	Prescription	Modality	Chemo Y/N Agent
Demers, 2014^1^	Colorectal Gynaecological (endometrium and cervix) Prostate	40–50.4 Gy 76 Gy (EBRT only. prostate + seminal vesicles)	EBRT modality not reported *Some gynae patients had brachytherapy pre‐ or post‐EBRT. Brachytherapy doses not reported	No (*n* = 108) Yes (*n* = 120) *Chemo information missing for one patient Cervical agent: cisplatin Colorectal agent: 5‐fluorouracil or capecitabine
Forslund, 2019^2^	Prostate	50 Gy/25#, + Boost 20–30 Gy/2‐10#	IMRT or VMAT Boost: brachytherapy, photon or proton *49 patients treated with a rectal retraction rod to reduce rectal wall dose	Adjuvant chemo (docetaxel) post RT (*n* = 8). NIG *n* = 6, SCG *n* = 2
Garcia‐Peris, 2016^3^	Gynaecological (cervix, endometrium, vulva–vagina, uterus)	52.2 Gy/29# (postoperative)	3DCRT (4‐field technique supine) 2DRT – 2‐field AP/PA prone) Brachy 1 week later for cervix/lymphovascular involvement	*n*
Linn, 2018^4^	Gynaecological (cervix)	50 Gy/25#	2DRT (AP/PA)	Concurrent chemotherapy: Placebo: *n* = 22 (78.6%), NIG *n* = 19 (73.1%)
Mansouri‐Tehrani, 2016^5^	Bladder Colorectal Gynaecological (endometrium, ovary, cervix) Prostate Other (bone sarcoma)	40–50 Gy	2DRT	Not clear
Petterson, 2012,^6^ 2014^7^	Prostate	50 Gy/25#^6^ + Boost 20 Gy/2‐4#	EBRT modality not reported HDR brachy boost (20 Gy) (*n* = 80) Proton boost (20 Gy) (*n* = 50)	NR
Ravasco, 2005^8^	Colorectal	50.4 Gy/28#	RT modality not reported	All Fluorouracil plus folinic acid
Soto‐Lugo, 2017^9^	Gynaecological (cervix, endometrium)	50 Gy/25# or 50.4 Gy/28#	3DCRT 4‐field technique	Cisplatin or carboplatin Concurrent chemotherapy: NIG *n* = 9 (69%), SCG *n* = 10 (77%)
Wedlake, 2017^10^	Colorectal Gynaecological (endometrium, cervix, vagina, vulva)	45–55.8 Gy	3DCRT or IMRT Gynaecological – received brachytherapy where indicated	Concomitant chemotherapy: 121 (72%) SCG *n* = 38 (69%) NIG groups: low *n* = 41 (75%), high n = 42 (75%) Agents: Colorectal: capecitabine Anus: IV mitomycin C plus capecitabine Cervix: cisplatin
Wedlake, 2012^11^	Bladder Colorectal Gynaecological (endometrium, cervix, ovary, vulva) Prostate	54 Gy (median) 36–74 Gy	EBRT modality not reported	Concomitant chemotherapy: 59 (50%) SCG *n* = 17 (44%) NIG (1) *n* = 18 (45%), (2) *n* = 24 (63%)
Weston, 2019^12^	Prostate	78 Gy/39#	EBRT modality not reported	NR

3DCRT, 3‐dimensional conformal RT; 2DRT, conventional 2‐dimensional; EBRT, external beam radiotherapy; Gy, gray; HDR, high‐dose rate; IMRT, intensity modulated radiation therapy; NIG, nutritional intervention group; NR, not reported; SCG, standard care group; VMAT, volumetric modulated arc therapy.

^1^Demers et al.^5^ ;^2^Forslund et al.^31^; ^3^Garcia‐Peris et al.^34^; ^4^Linn et al.^32^; ^5^Mansouri‐Tehrani et al.^35^; ^6^Pettersson et al.^26^; ^7^Pettersson et al.^29^; ^8^Ravasco et al.^27^; ^9^Soto‐Lugo et al.^36^; ^10^Wedlake et al.; ^11^Wedlake et al.^28^; ^12^Weston et al.^37^

### Nutritional interventions

All 11 studies involved DC in the intervention. Seven included a supplement in addition to DC, and four studies included DC only. Supplements used included probiotics (*n* = 3),^5^, ^32^, ^35^ prebiotics (*n* = 1),^34^ probiotic + soluble fibre (*n* = 1),^37^ high protein liquid supplement (*n* = 1)^27^ and fat emulsion (*n* = 1)^28^ (Table 3).

**Table 3:** Study and intervention characteristics.

Author (year)	Country	Study characteristics		Participants		Arms		Compliance Compliance
		Study type (number of arms)	*n*	Sex (% F)	Intervention	Control	Both arms receiving DC	Compliance of dietary intervention reported
Demers, 2014^1^	Canada	RCT (*n* = 3)	229	50	Probiotics (high‐dose group and standard‐dose group)	Placebo and DC	Yes	Yes
Forslund, 2019^2^	Sweden	RCT (*n* = 2)	157	0	Diet: reduce lactose and replace insoluble with soluble fibres	Habitual diet	IG only	Yes
Garcia‐Peris, 2016^3^	Spain	RCT (*n* = 2)	38	100	Prebiotics	Placebo and DC	Yes	Unclear
Linn, 2018^4^	Myanmar	RCT (*n* = 2)	54	100	Probiotics	Placebo	Yes	Yes
Mansouri‐Tehrani, 2016^5^	Iran	RCT (*n* = 3)	67	42	Probiotics (with and without honey)	Placebo plus yoghurt	Yes	Unclear
Petterson, 2012,^6^ 2014^7^	Sweden	RCT (*n* = 2)	113	0	Diet: reduce lactose and replace insoluble with soluble fibres	Habitual diet	IG only	Yes
Ravasco, 2005^8^	Portugal	RCT (*n* = 3)	111	41	Individualised DC vs high‐protein liquid supplement	Habitual diet	IG only	Yes
Soto‐Lugo, 2017^9^	Mexico	RCT (*n* = 2)	26	100	Diet: FODMAP	Habitual diet	IG only	Yes
Wedlake, 2017^10^	United Kingdom	RCT (*n* = 3)	159	58	Diet: modify fibre intake (low vs high fibre)	Habitual diet	IG only	Yes
Wedlake, 2012^11^	United Kingdom	RCT (*n* = 3)	117	32	Diet: low or modified fat diet (low fat diet vs modified fat diet with fat emulsion)	Normal fat diet prescribed, LCT dietary fats calculated to comprise 40% of total energy	Yes	Yes
Weston, 2019^12^	Australia	RCT (*n* = 2)	17	0	Soluble fibre (Metamucil) + probiotics with low gas diet	Standard care: Movicol half strength and low gas diet	Yes	Unclear

RCT, randomised control trial; DC, dietary counselling; F, female; IG, intervention group; LCT, long‐chain triglycerides.

^1^Demers et al.^5^ ;^2^Forslund et al.^31^; ^3^Garcia‐Peris et al.^34^; ^4^Linn et al.^32^; ^5^Mansouri‐Tehrani et al.^35^; ^6^Pettersson et al.^26^; ^7^Pettersson et al.^29^; ^8^Ravasco et al.^27^; ^9^Soto‐Lugo et al.^36^; ^10^Wedlake et al.^33^; ^11^Wedlake et al.^28^; ^12^Weston et al.^37^

Of the 11 studies, only one involved individualised dietary counselling as the intervention.^27^ Six studies provided DC to both intervention and control, in which all but one study provided the same advice across the groups.^5^, ^28^, ^32^, ^34^, ^35^, ^37^ In the remaining studies in which DC was provided to only the intervention arms, control groups were advised to maintain habitual dietary intake (with or without the use of supplements) (*n* = 5). The DC advice varied between studies. Wedlake et al.^28^ prescribed differing amounts and types of fats. In two studies, patients were advised to avoid insoluble fibre and lactose.^26^, ^31^ Advice provided in other studies included instruction to avoid lactose and fermented foods and follow a low‐fat, low‐fibre diet (*n* = 1)^34^ or given individualised targets for energy and protein (*n* = 1),^27^ fat (*n* = 1),^28^ or fibre, carbohydrate and fat (*n* = 1).^5^ Three studies prescribed diets, including a low FODMAP diet,^36^ low gas diet^37^ and a high fibre diet.^33^ The remaining two studies provided little detail on the DC component. Linn et al.^32^ provided a pamphlet of standard dietary advice, and Mansouri‐Tehrani et al.^35^ provided a list of allowed and prohibited foods, including prohibiting dairy other than the prescribed yoghurt. DC was delivered via sessions with a dietician, which at times were supported with written resources (*n* = 7).^5^, ^26^, ^27^, ^29^, ^31^, ^33^, ^37^ or exclusively by written mediums only such as pamphlets or booklets (*n* = 4).^28^, ^32^, ^35^, ^36^ In one study, it was unclear how DC was delivered.^34^ In the studies involving contact with a dietitian, DC was administered weekly throughout RT,^19^, ^25^, ^28^ and three studies provided DC at the start, during RT and post‐RT completion.^26^, ^29^, ^31^, ^33^ The active duration of the interventions ranged from four weeks to 26 months.

### Gastrointestinal toxicities

All articles utilised internationally clinically accepted and validated assessment tools to assess and record toxicities. All articles, bar one,^32^ engaged more than one assessment tool. The most common tools utilised to record GI toxicities was the EORTC QLQ‐C30 (n = 5),^26^, ^27^, ^29^, ^31^, ^36^ including the tool’s additional disease‐specific modules for prostate (PR25), cervical (CX24) and endometrial cancers (EN24); and the CTCAE versions 2.0, 3.0 and 4.0 utilised in five articles to report toxicity incidence and severity.^5^, ^32^, ^34^, ^35^, ^36^ The Radiation Therapy Oncology Group and EORTC Radiation Morbidity Scoring Criteria were utilised in two articles.^27^, ^28^ A Gastrointestinal Side Effects Questionnaire (GISEQ) was employed in two studies.^26^, ^29^, ^31^ The Inflammatory Bowel Disease Questionnaire (IBDQ) was used in two articles.^28^, ^33^ Five articles utilised the Bristol scale for reporting stool consistency.^5^, ^28^, ^33^, ^34^, ^35^

In total, 104 individual toxicity‐related outcomes were reported across the 11 studies (Table 4). As several studies utilised more than one assessment tool to assess the same toxicity, some outcomes were counted more than once within the same study.

**Table 4:** Outcomes reported.

Outcome category	Times reported	Articles reported (*n*)
Diarrhoea	24	11
Pain and or cramping	15	9
Bloating and/or flatulence	11	5
Constipation	9	6
Nausea and or vomiting	7	3
Combined symptom scores	7	5
Faecal incontinence and/or leakage	5	5
Blood in stool	5	3
Anorexia and or loss of appetite	4	3
HRQOL overall score	4	3
Fatigue	3	2
Limitations on daily activities	3	3
Mucus	3	3
Proctitis	1	1
Use of medication required (unspecified)	3	3

The most common toxicities reported were diarrhoea, pain/cramping, bloating/flatulence and constipation. In five articles, outcomes were also recorded as combined symptom scores, in which individual toxicities were grouped together to provide an overall score.^26^, ^28^, ^29^, ^33^, ^36^

#### Diarrhoea

Diarrhoea was assessed as an outcome in 11 articles^5^, ^26^, ^27^, ^29^, ^31^, ^32^, ^33^, ^34^, ^35^, ^36^, ^37^ and included in an overall grouped symptom score in one article.^28^ Three studies found a reduction of diarrhoea incidence.^27^, ^32^, ^35^ Linn et al.^32^ reported reduced incidence in the intervention group receiving probiotics (53.8 vs. 82.1%, *P* < 0.05), whilst also reporting a decrease in severity (*P* < 0.05). Mansouri‐Tehrani et al. also utilised probiotics in their intervention groups and reported a decrease in daily stool movements, diarrhoea mean grade and reduced need for antidiarrhoeal medication.^35^ Ravasco et al.^27^ reported rates of diarrhoea higher in the standard care group at the end of RT and three months post‐completion compared with groups receiving a high‐protein liquid supplement or individualised dietary counselling.

#### Constipation

Constipation as an outcome was reported in six articles.^26^, ^27^, ^29^, ^31^, ^34^, ^36^ Nil significant differences in outcomes were found in any of these studies.

#### Pain/Cramping

Pain and/or camping was reported in 9 articles.^5^, ^26^, ^27^, ^29^, ^31^, ^32^, ^34^, ^35^, ^36^ Linn et al. reported a reduction in Grade 2 abdominal pain incidence in the probiotic group (3.8%) compared with the placebo group (57.1%, *P* = 0.000). This was also reflected in a reduction in the number of reported days of abdominal pain occurrence.^32^

### Quality of life

The EORTC QLQ‐C30 and the site‐specific modules PR25, CX24 and EN‐24 were used in seven articles (six studies) to assess individual GI toxicities and overall QOL scores.^5^, ^26^, ^27^, ^29^, ^31^, ^34^, ^36^ Ravasco et al. reported a significant improvement in median QOL function scores within the individual DC group at RT completion (*P* < 0.002).^27^ These improvements were proportional to increases in intake of energy and protein, and nutritional status improvement (*P* < 0.05). The two studies by Wedlake utilised IBDQ scores to assess QOL.^28^, ^33^ Wedlake’s 2017 article reported a significant difference in IBDQ score reduction between baseline and end RT completion within the intervention group receiving a high‐fibre diet and the habitual diet group (*P* = 0.015).^33^

#### Nutritional status

Nutritional status was assessed in three studies^27^, ^29^, ^31^ using the Scored Patient‐Generated Subjective Global Assessment (PG‐SGA), however, was only reported by Ravasco et al., who also used BMI as another measure of nutritional status to identify malnutrition. The intervention group receiving individualised DC had significantly fewer patients deteriorating in nutritional status at both the end of RT and at three months, using PG‐SGA and BMI scores (*P *< 0.001). The protein supplement and the habitual diet groups presented with significantly more nutritional deterioration, in both incidence and severity, at the end of RT and three months post‐treatment (*P *< 0.001). At three months post‐RT, the individualised DC group was the only group to have patients malnourished at baseline (nine of 15) to improve PG‐SGA scores, with an average gain of 4 kg. Additionally, improvements in nutritional status positively correlated with QoL scores (*P* < 0.5).

#### Weight loss

## Four studies reported on changes in weight; however, nil reported a significant change in weight loss between control and intervention groups.^27^, ^28^, ^33^, ^36^

## Discussion

This review set out to assess and evaluate current nutritional interventions involving dietary counselling and their effect on gastrointestinal toxicities, weight loss and QOL arising in patients receiving pelvic radiotherapy. Compared with previous reviews conducted assessing the effectiveness of NI, our review focuses on interventions that included dietary counselling. Of the 11 studies included in this review, only one paper involved individualised DC as an intervention.^27^ Most nutritional interventions used in this review were not well‐described. Given the few studies identified and included in this review, as this is a growing area of research, it is unclear which is the best practice approach for the nutritional management of GI toxicity; hence the studies included in this review are quite varied in their nutritional approach.

The management of GI toxicities in radiotherapy is vitally important due to the impact toxicities may have on treatment adherence and completion. Pauses or incompletion of scheduled RT treatments due to toxicities may influence overall treatment effectiveness. In a retrospective analysis of 1227 patients receiving curative RT for a variety of disease sites, it was reported that patients who miss two or more treatments are at an increased risk of recurrence (5‐year cumulative incidence 16 vs. 7%, *P* < 0.001) and have inferior recurrence‐free survival (5‐year actuarial rate 63 vs. 79%, *P* < 0.001) and inferior overall survival (5 year OS 72 vs. 83%, *P* < 0.001).^38^ Given the documented role of diet in GI toxicities and associated dietary intake during RT, the effectiveness of nutritional interventions was synthesised in this review.

### Summary of key findings

Nutritional interventions were reported to be effective in reducing diarrhoea in three articles. Two interventions involved probiotics taken throughout RT treatment, and the third article by Ravasco et al. had improvements in both individualised dietary counselling and the high‐protein liquid supplement intervention groups. Constipation was reported in six articles; however, nil significant differences were identified in any of these studies. Quality of life improvement was reported in three of eight studies that had overall QOL as an outcome. The sole intervention reporting an improvement in overall QoL scores between baseline and RT completion was individualised DC.^27^

It is important to note the self‐reporting nature of the outcome tools utilised to assess GI toxicities and QOL, which has the potential to lead to subjective variations between participants and studies. Given the limited studies, these results are not generalisable to broader groups and ethnicities, but results agree with the previous systematic review by Lawrie^24^ that showed a need for further high‐quality studies powered to detect a change.

### Dietary counselling

The one study assessing individualised DC reported positive outcomes in nutritional status, diarrhoea and QOL scores. Ravasco’s results reported at RT completion and at three‐month follow‐up, the DC IG experienced an improvement in all QOL function scores (including physical, emotional, cognitive, social, role and global health or QOL) proportionally with adequate dietary intake (*P* < 0.05 and *P* < 0.02). In comparison, at RT completion, the habitual diet group QOL function scores decreased (*P* < 0.05). The DC IG also performed better than the protein supplement group, which had improved in only three of six function scores (physical, role and emotional) (*P* < 0.05). The protein supplement group either maintained or decreased their QOL scores (*P* < 0.03), whereas the habitual diet group had decreased in QOL at the end of RT (*P* < 0.004). A positive correlation was present in all groups with regard to dietary intake, nutritional status, and QOL function scores. The DC IG‐positive QOL scores were associated with adequate dietary intake (*P* < 0.01) and nutritional status (*P* < 0.02), whereas the habitual diet group had a strong association between declining QOL function scores and poor dietary intake (*P* < 0.001) and nutritional status (*P* < 0.002).

The current DAA guidelines suggest that individuals undergoing radiotherapy be referred to or screened for dietary assessment. Six of the eleven studies reported frequent contact between patients and dietitians to provide dietary information.^5^, ^27^, ^29^, ^31^, ^33^, ^37^ In other studies, dietary information was provided via written mediums only, which shows the advice may not have been personalised and could therefore be provided by a range of health professionals such as RTs. This is beneficial compared with offering nil dietary support; however, given the evolution of dietetic practice, future studies could include providing more personalised advice that may further improve GI toxicities. The DAA recommendations entail using validated nutrition assessment tools, such as the scored Patient Generated‐Subjective Global Assessment (PG‐SGA), to assess the nutritional status of patients receiving RT. The nutrition intake recommendation of the DAA aims for an energy intake of 125 kJ/kg/day and at least 1.2 g protein/kg/day. However, it was shown that only two studies attempted to ensure that patients followed this guideline or similar. The DAA guidelines were first published in 2008;^39^ hence, available before 11 of the studies were published. Given the availability of these recommendations, this can be considered when interpreting the findings.

As the studies included in this review held a high degree of heterogeneity, a meta‐analysis of the outcomes is not possible. Varying factors between the articles included different tools used to assess toxicities, different time points and different measures reported such as incidence, prevalence, proportion and severity.

Control groups varied between the articles as five of the studies did not provide any DC to the control groups, in which participants kept to their habitual diet.^26^, ^27^, ^28^, ^29^, ^31^, ^33^, ^36^ The remaining six studies provide DC to the control arm in addition to either a placebo or control measure such as Movicol. Of the five studies with habitual diet as the control with nil DC, one study used individualised DC as the intervention,^27^ and four studies utilised standardised dietary counselling based on specific nutrient intake guidelines, for instance, fibre or FODMAP diet. A relatively small number of the studies assessed the interventions against habitual diet as the control arm, making it difficult to attribute the results of the study to the intervention alone.

### Quality of life

The EORTC QLQ‐C30 was the most utilised tool amongst the included studies. Ravasco et al. reported an improvement in all QOL function scores (*P* < 0.05), including physical, emotional, cognitive, social, role and global health, at RT completion in the individualised DC group. In comparison, within the habitual diet group, all QOL function scores decreased (*P* < 0.05). In the Soto‐Lugo study, at RT completion, the FODMAP intervention group had significantly lower QOL symptom scores in the cervical subset (CX‐24) group. However, this was also not represented in the general QLQC‐30 or endometrial (EN‐24) questionnaires.^36^ In the Wedlake 2017 article, a significant difference of IBDQ scores between baseline and end of RT was reported between the habitual diet and high‐fibre diet groups. The high‐fibre group reported a smaller score decrease; however, there was no significant difference against the low‐fibre group or between the low versus habitual groups. At 1‐year post‐RT, IBDQ scores improved compared with baseline in the high‐fibre group yet reduced in the habitual group.

#### Weight loss

The monitoring of WL during RT is essential; however, it can be challenging to ascertain if weight changes result from the effectiveness of an NI or due to the aggressive biological nature of cancer. Weight loss is considered critical if WL >5% from the start of RT until week 8 or >7.5% until week 12.^40^ Weight loss has the potential to alter a patient’s external contour, which may lead to a geographic miss or changes in dose delivered to target volumes or OARs, thus influencing treatment effectiveness.^17^, ^18^ The tolerance of acceptable WL is dependent on the anatomical site being treated, and the treatment technique used as the dosimetric effect of WL is intensified in highly conformal RT techniques such as IMRT or VMAT.^18^ In VMAT prostate plans, D’Souza et al. have shown that the maximum dose to the planning target volume can change by 3.7–4.1% per centimetre lost due to WL.^19^ Caution must be taken in such scenarios to ensure the patient’s initial treatment plan remains achievable without compromising on dose to target volumes and critical organs.^17^ Although weight loss can result from GI toxicities impacting nutritional intake, only four studies assessed weight loss, with nil reporting a significant change.^27^, ^28^, ^33^, ^36^

#### Sample sizes

Overall, the sample sizes of each RCT were small (17‐229). In comparison with other research areas, these numbers are considered small, limiting the generalisability of the findings of this review.

#### Intervention delivery

In this review, studies included brief descriptions of what each DC component entailed, for example, the information and advice provided to participants. Additionally, the intervention delivery methods (e.g. if delivered face‐to‐face or written) were mostly overlooked in the articles. When both control and intervention groups were prescribed the same diet, the majority of articles did not state clearly if this was implemented as a control measure to homogenise the groups when assessing the use of supplements. For patients who have access to dietetic support, the effectiveness of nutritional interventions is limited by patient compliance and may vary whether the information delivered is individualised or a generically published informational pamphlet. Of the 11 studies, eight reported on compliance to the nutritional intervention.

#### Radiotherapy techniques

Some studies^32^, ^34^, ^35^ utilised outdated treatment techniques that are not congruent to modern global standards. IMRT or VMAT, where this practice is available, is the preferred optimal treatment modality for pelvic radiotherapy due to increased dose conformity and normal tissue sparing. Outdated techniques deliver a higher dose to the organs at risk (OARs), particularly the rectum and bowels, which may increase the risk of GI toxicity development. Additionally, nil articles referred to the imaging protocol and whether soft tissue matching was utilised – a technique used to ensure the target is located within the target volumes and sensitive organs are not exposed to additional unnecessary irradiation. Zero articles reported on the treatment plan quality or provided a dose‐volume histogram (DVH) or data regarding dose constraints to organs at risk; however, Soto‐Lugo reported on rectum dose a constraint of 40 Gy > V60%.^36^ By not considering the dose received by OARs, it is difficult to assess the effectiveness of NIs in decreasing gastrointestinal toxicities entirely. A patient is more at risk of experiencing toxicities if an OAR receives an amount of radiation that exceeds the recommended constraint. Patients are also more susceptible to radiation toxicities and morbidity if also receiving concurrent chemotherapy.^6^ Demers was the only article to produce a subgroup analysis by stratifying outcomes by surgery status. Significant differences were found between the groups as patients who received surgery before RT experienced more severe diarrhoea than those who had not had surgery. In the same subgroup, the standard dose probiotic intervention was more effective in reducing diarrhoea incidence (*P* = 0.05) than in patients who did not have surgery (*P* = 0.66).^5^

To wholly compare and interpret the effectiveness of nutritional interventions, data regarding dose received by OARs and the stratification of results by concurrent or adjuvant chemotherapy and presurgical status are required.

## Conclusion

This review has demonstrated a lack of published RCTs exists on nutritional interventions focusing on individualised dietary counselling for pelvic radiotherapy patients in the modern context. Studies confirming the role of nutritional interventions in radiotherapy for head and neck patients are widely published; however, comparatively for the cohort of pelvic cancers, the research is lacking.

Given the significant impact that gastrointestinal toxicities have on patients undergoing pelvic RT, further studies assessing the effectiveness of dietary counselling interventions are required, preferably in the context of modern radiotherapy treatment techniques and prescriptions with attention to OAR dose reporting. The stratification of concurrent or adjuvant chemotherapy and presurgical status should also be considered when evaluating outcomes.

## Conflict of interest

The authors declare no conflict of interest.
